# The Ethics of Decentralized Clinical Trials and Informed Consent: Taking Technologies’ Soft Impacts into Account

**DOI:** 10.1007/s10728-024-00483-1

**Published:** 2024-05-19

**Authors:** Tessa I. van Rijssel, Ghislaine J. M. W. van Thiel, Johannes J. M. van Delden, On behalf of the Trials@Home consortium

**Affiliations:** Department of Bioethics and Health Humanities, Julius Center for Health Sciences and Primary Care, University Medical Center Utrecht, Utrecht, The Netherlands

**Keywords:** Research ethics, Technology ethics, Digitalization, Clinical trials, Informed consent

## Abstract

Decentralized clinical trials (DCTs) have the potential to advance the conduct of clinical trials, but raise several ethical issues, including obtaining valid informed consent. The debate on the ethical issues resulting from digitalization is predominantly focused on direct risks relating to for example data protection, safety, and data quality. We submit however, that a broader view on ethical aspects of DCTs is needed to touch upon the new challenges that come with the DCT practice. Digitalization has impacts that go beyond its direct purposes, by shaping behaviors, experiences, social relations, and values. We examine four elements of the informed consent procedure that are affected by DCTs, while taking these soft impacts of technologies into account: (i) informing participants and testing understanding, (ii) freedoms in relation to responsibilities and burdens, (iii) trust in participant-researcher relations, and (iv) impacts on the concept of privacy. Our analysis reveals that a broad view is key for optimal conduct of DCTs. In addition, it provides insight into the ethical impacts of DCTs on informed consent. Technologies such as DCTs potentially have profound impacts which are not immediately addressed by the existing regulatory frameworks, but nonetheless important to recognize. These findings can guide future practices of DCTs to foster the important values of clinical research in this novel approach for conducting clinical trials.

## Introduction

Decentralized clinical trials (DCTs) are technology-enhanced clinical trials, that move trial activities from clinical settings to participants’ immediate surroundings. DCTs promise to be more efficient, allow collection of larger amounts of data (e.g., through apps and wearables), and to improve recruitment, retention, and diversity of study populations. DCTs could therefore generate more representative data, a nd may contribute to more freedom, flexibility, and empowerment for participants in research [12, 19, 37].

As DCTs alter and disrupt current practices of research, they raise ethical issues. While DCTs, and digitalization in general, are becoming increasingly common, it is important to promote a responsible practice–especially with regards to core ethical requirements such as informed consent. Obtaining valid informed consent has been flagged as an important ethical challenge when using decentralized methods in clinical trials [7, 41, 42, 44]. In the traditional clinical trial practice, informed consent procedures consist of an in-person conversation, in which successful communication and participant-researcher relationships are needed [24]. DCTs raise therefore ethical questions concerning the relevance of in-person contact and the impacts of digitalization on this specific practice. While DCTs can also include in-person contacts such as home visits by nurses, we focus on an informed consent process that is fully replaced by digital tools. Such an informed consent procedure could involve several steps in practice, including informing participants through smartphone applications or videos, enabling researcher-participant interactions through (video)calls, and online identity verification systems and electronic signatures for signing consent forms.

Questions surrounding the necessity for in-person contact in informed consent procedures implicitly refer to the so-called ‘soft impacts’ of technologies. These are impacts of technologies and digitalization that are more difficult to grasp, such as impacts on communication and social relations [38]. Although the current regulatory frameworks do not directly address these aspects, recognizing soft impacts is essential for achieving a comprehensive understanding on the impacts of digitalization.

In this paper, we argue that a broader view on the ethical aspects of digitalization in clinical trials is needed. Technologies’ soft impacts need to be recognized when implementing new, possibly disruptive technologies, such as DCTs. To illustrate this, we analyze how soft impacts of DCTs, related to having no in-person contact, may shape the practice of informed consent, using the existing literature and empirical evidence. This demonstrates that a broader view on the ethical aspects of digitalization in clinical trials is needed, while also providing insight into the validity of informed consent in DCTs.

## Digitalization and Technology Ethics

The current debate on ethical aspects of digitalization in clinical trials often focusses on the direct risks associated with digital technologies, such as risks relating to data protection, safety, and data quality [7, 14, 40]. While these aspects remain important, other ways in which new technologies shape existing practices, may be overlooked [45–47]. This especially concerns impacts that are more difficult to quantify, such as impacts on social relations, values, or behaviors and experiences. These types of ‘soft’ impacts of technology are more qualitative, and difficult to measure, compared to the more direct and tangible ‘hard’ impacts and risks related to technologies. Moreover, as opposed to hard impacts, soft impacts are generally also shaped by users’ behavior and interaction with novel technologies, and it is not always immediately clear whether these impacts are (un)desirable [38]. These soft impacts and changes of practices that result from novel technologies such as DCTs are difficult to predict. Because DCTs are not (yet) fully developed and used widely, it is difficult to foresee their precise impact on research practices. Anticipating these soft impacts is however essential for responsibly and successfully implementing new, possibly disruptive, technologies, instead of having to deal with possible undesirable effects of technologies once they appear [38, 48].

The philosophy of technological mediation focuses on ways in which practices may be altered by digitalization. Technologies are not considered to function merely as neutral tools but are instead able to actively mediate the way we perceive reality and create new practices. They highlight certain aspects of reality and make certain types of behavior more likely than others. On the other hand, designers and users of technologies can also actively shape new technologies, which offers opportunities when introducing new technologies such as DCTs [45, 46]. Technological mediation implies that the ethics of technology should not consider new technologies as a threat, that we either have to accept or reject. Instead, ethics should focus on the question of *how* novel technologies can be used in a responsible manner, by anticipating the possible implications of specific technologies, which values may be at stake, and how interpretations of existing values may change [48].

The questions surrounding the necessity for in-person contact in the informed consent procedures and the impacts of digitalization in DCTs predominantly relate to soft impacts of technologies. Therefore, we use the concept of soft impacts to analyze DCTs’ impact on informed consent procedures. The philosophical approach of technological mediation–combined with the available empirical research on impacts of existing technologies–enables anticipating plausible soft impacts of digitalization on practices of clinical research and informed consent [38].

## Informed Consent in Clinical Research

Informed consent is one of the core ethical principles for clinical research and a legal requirement for conducting research with participants. Informed consent procedures are essential in fostering and respecting participants autonomy and to protect participants from harm. A valid informed consent for a clinical study entails an autonomous authorization to participate in a study, based on comprehension of relevant information and in the absence of undue influence [3, 4].

Informed consent consists of three important elements [3, 4]. First, it has to be determined that a potential participant is capable of giving informed consent. A lack of ability to understand, communicate, reason or deliberate on the part of the participant inhibits a voluntary and informed decision to participate in research [4]. Secondly, the Declaration of Helsinki states that participants need to be adequately informed on *“the aims, methods, sources of funding, any possible conflicts of interest, institutional affiliations of the researcher, the anticipated benefits and potential risks of the study and the discomfort it may entail, post-study provisions and any other relevant aspects of the study”* [49]. In practice, it is often required that prospective participants receive this information in written form. Subsequently, an interview with a qualified member of the research team should take place in which participants should have the opportunity to ask questions, and the researcher is responsible for verifying whether a participant is competent and has sufficient understanding of the information, as is for example specified in article 29 of the 536/2014 EU Clinical Trials Regulation (CTR). This in-person contact is often deemed to be an essential aspect of informed consent processes [10, 11]. Thirdly, consent needs to be voluntary, which implies that a participant’s decision needs to be free of controlling influences. A range of possible controlling and non-controlling influences exists. For example, when a participant is recruited for research by their treating physician, the existing relation between them could be experienced as a controlling influence. Intentional manipulation by a physician by framing information in a certain way is an example of a graver form of controlling influence. However, there is no sharp distinction between controlling and non-controlling influences [26]. Finally, a prospective participant has to be informed on their right to refuse or to withdraw their consent [49].

Ideally, informed consent does not end with signing an informed consent form, but instead is a continuous process of keeping participants informed, engaged, and motivated to participate in a clinical study. It starts at the first contact with a participant and continues throughout the study [13, 23].

## Impacts of Digitalization on Informed Consent

We identified four aspects of DCTs and decentralized informed consent procedures that are impacted by digitalization, based on a previous study in which research ethics committee members reviewed a DCT protocol [42], and on an analysis of the relevant literature and the existing empirical evidence. These four aspects are, (i) informing participants and testing understanding, (ii) freedoms in relation to responsibilities and burdens, (iii) trust, and (iv) privacy.

### Informing Participants and Testing Understanding

Informing participants and testing their understanding is an essential part of informed consent procedures [4, 49]. Evidence suggests that understanding is often incomplete in current informed consent processes [16, 17]. Simply translating current practices–which usually involve long and complex documents–to a digital form would thus not be desirable [16, 17].

Here, altering informed consent processes with new technologies may provide opportunities for improvement [13]. Digital platforms could for example adapt to participants’ individual needs [8, 35]. Participants could be enabled to control the amount of information they receive and to decide when, where and how they want to receive information (e.g., going through instruction materials multiple times, taking breaks, or inviting others for advice). Additionally, multiple studies suggest that participants feel better informed through for example video’s, interactive features, or gamification, instead of conventional informed consent forms. Especially combining multiple ways of presenting information (e.g., audio, video, graphics) seems to have a positive impact [15, 22, 35]. Incorporating these diverse formats, and for example language translations, can also promote inclusivity, although it should be noted that the digital format also poses barriers.

It has been questioned if digital contact is sufficient for assessing a potential participants’ competency, understanding and voluntariness. The lack of in-person contact may bring challenges for these interactive parts of informed consent procedures [2, 22]. Researchers have for example experienced difficulties in checking participants’ understanding adequately in DCTs [9]. A lack of body language may hinder communication, which makes it more difficult for researchers to notice hesitancy or incomprehension in participants. Research on digital health practices has suggested that healthcare professionals usually rely on visual cues (e.g., body language) and in-person contact to get an impression of patients’ health and to build relationships, but that the physical distance causes them to rely more on listening to patients and asking the right questions, which requires specific communication skills [30].

### Freedoms in Relation to Responsibilities and Burdens

In general, the DCT approach gives more flexibility and freedoms to participants. Trial activities that are normally carried out on-site under supervision of research staff are moved to the participant’s home. Recruitment, enrolment, and informed consent procedures taking place outside the traditional healthcare context may positively impact the voluntariness of informed consent. The question to participate–especially by a treating physician–can bring a certain degree of social pressure, depending on the specific (cultural) context [10]. In addition, a potential participant may experience less (time-)pressure to make a quick decision without fully understanding the relevant information or without having additional discussions with relatives or friends [1]. And finally, the use of digital platforms could make revoking consent as well as reconsenting easier for participants [8].

This freedom and flexibility however also implies that participants themselves become more responsible for being informed and for executing trial-related activities. Activities such as the verification of identity and diagnosis, a valid signature, and the checking of inclusion- and exclusion criteria are less self-evident in DCTs than in site-based clinical trials [17]. This places an additional burden on participants, as they have to demonstrate that they are trustworthy and suitable for participating in the trial, for example through online identity verification systems and providing access to their medical records. Moving all trial activities to a participants’ home thus unintentionally shifts certain responsibilities and possible burdens to participants, which requires not only a level of digital (health) literacy, but also a form of self-disciplining [25]. Furthermore, it should be noted that requiring a certain level of digital (health) literacy may unintentionally exclude groups from participating in DCTs [42].

### Trust

A certain level of trust is needed to overcome differences in the level of knowledge and understanding between researchers and trial participants in the process of informed consent [20, 33]. Trust is a form of social capital that is based on transparent communication and building relationships [21, 33]. The lack of in-person contact, physical distance, and perceived anonymity in the digital environment, may impact social relations, and building trust during informed consent procedures may therefore be more difficult [42]. This might especially be the case for populations with already more distrust in healthcare and research institutions [31, 36]. DCTs could thereby exacerbate barriers to participation for these populations. In addition to this, the use of digital technologies in DCTs can bring additional (e.g., privacy-related) risks, which may cause distrust among prospective participants. Furthermore, the motivation to participate in a clinical trial may come less naturally, due to the lack of existing relations and familiar institutions [42]. For example, the recruitment and enrollment of participants does not happen through a known and trusted healthcare provider or the institution that they represent. This may negatively impact the willingness to provide informed consent [10].

Similarly, it should be noted that researchers’ trust towards participants may also come less naturally. This may place a burden of proof of trustworthiness on the participant. In addition to online identity verification systems, researchers can use technologies to monitor participants’ reliability and compliance to a greater extent in DCTs as compared to traditional trials. This is for example the case with systems to remotely monitor participants’ intake of medication. The added value of these technologies for patients is questioned, while these technologies can be disproportionately intrusive and may impact the participant-researcher relationship negatively [43].

It is often emphasized that informed consent should be a process, instead of a single moment of signing an informed consent form [13, 23]. Here, the use of a digital platform or digital communication tools can offer opportunities, especially since previous research in the context of digital healthcare has suggested that frequent communication through for example phone calls is important to build a trusting relationship with patients [30]. More frequent communications and multiple ways of interaction through for example digital platforms could therefore improve decentralized informed consent processes and may compensate for the lack of in-person contact [17].

### Privacy

Privacy in the context of DCTs is primarily concerned with data protection and having control over personal information. The use of apps and devices increases the risk of passive collection of audio, video, or location tracking data, and the possible data sharing with commercial parties [5, 7, 14]. Participants therefore need to be informed to a greater extent on what data is being collected and who has access to their data, due to, among others, more complex data flows [5, 7, 14]. However, the distinction between public and private spheres has also shaped the concept of privacy to a large extent [27, 28] The fact that the participants’ own home becomes the research site in a DCT means that this distinction can become unclear, which may also cause privacy-related issues. For example, informed consent processes taking place through video calls, implies that participants are being videorecorded in their homes and that it can be unclear who has access to the information that is shared during these video calls.

Defining privacy as contextual integrity provides more insight into how privacy is impacted by digitalization. Instead of focusing on the distinction between public and private or personal information, and the protection of personal information, contextual integrity focusses on the appropriateness of information flows in multiple contexts. Different social contexts are governed by informational norms and expectations on what information is appropriate to share with whom, and according to contextual integrity, these contexts should determine restrictions on the flow of information [27, 29].

This perspective indicates two aspects in which DCTs impact the concept of privacy. First, as the examples above demonstrate, DCTs bring together multiple contexts, such as the healthcare context, participants’ own surroundings, but also commercial parties which are often involved in the apps and devices that are used. This causes a possible unclarity about which informational norms are leading, and participants being unaware of which parties have access to data [34]. Secondly, new technologies introduce new aspects to consider and enable actions for which no norm has been developed. New types of information are gathered or generated, other ways of dissemination become possible, and other actors are involved. This means that existing informational norms for specific contexts, such as the context of clinical research, may not be sufficient for guiding these new, technology-mediated, practices [28, 29].

## Conclusions

In this paper, we provide insight into potential impacts of the introduction of DCTs on informed consent, that go beyond current ways of analyzing these impacts. Although soft impacts are difficult to measure or predict and not immediately addressed by the existing regulatory frameworks, it is nonetheless important to monitor and address them. For example, trust is fundamental for informed consent and participation in clinical trials, and thus for implementing DCTs successfully. While we highlighted informed consent and used it to illustrate how the concept of technological mediation can help examine these impacts more closely, this analysis broadens the perspective on ethical aspects of DCTs in general. Relational aspects between researchers and participants, such as trust and communication, play a role throughout trials. Moreover, the transfer of responsibilities towards participants is likely to be present to an even greater extent during other trial activities, such as data collection.

As we described, DCTs are likely to mediate several aspects of informed consent procedures, and the relationships and communications between researchers and participants in general. There are several ways in which these insights could be integrated in future practices of DCTs, in order to foster the important values of clinical research. The advantages regarding the flexibility and opportunities that digital tools offer, should be utilized to optimize participants’ understanding, especially with regards to complex information (e.g., information related to data flows). Other challenges can be addressed through specific choices in the design of electronic consenting technologies and procedures. A good example of a technology that explicitly aims to evoke desirable practices and foster values such as autonomy is dynamic consenting, which has been originally developed for biobanks. Dynamic consent is a digital approach for consent procedures that gives participants the ability to update their consent preferences over time and control the usage of their data [6, 39]. A similar approach for DCTs could be suitable to adjust for the loss of in-person contact and foster relations and trust between researchers and participants. For example, a digital platform could promote more ongoing interactions and involvement of participants throughout trials. This would offer substantial advantages over traditional informed consent procedures, which occur mainly at the start of a trial. It should also be considered in which ways this could cause a larger burden on participants, by for example overloads of information or choices [32]. However, dynamic consent can also enable participants to control the amount of information they receive [18].

To conclude, we argue that a broader view on the ethics of digitalization in clinical research is needed. On the one hand, these technologies are able to improve the conduct of trials, by for example enhancing understanding, voluntariness, and participant satisfaction in informed consent procedures. On the other hand, we demonstrated how DCTs may have consequences on aspects that go far beyond their direct purposes, such as the effects on shifting responsibilities, existing relationships within the healthcare and research context, and the concept of privacy. For DCTs to be a valuable addition to clinical trial conduct, these soft impacts of technologies need to be recognized and addressed in the design and implementation of this novel approach for clinical trials.
